# Temporal window for detection of inflammatory disease using dynamic cell tracking with time-lapse MRI

**DOI:** 10.1038/s41598-018-27879-z

**Published:** 2018-06-22

**Authors:** Max Masthoff, Sandra Gran, Xueli Zhang, Lydia Wachsmuth, Michael Bietenbeck, Anne Helfen, Walter Heindel, Lydia Sorokin, Johannes Roth, Michel Eisenblätter, Moritz Wildgruber, Cornelius Faber

**Affiliations:** 1 Translational Research Imaging Center, Department of Clinical Radiology, University Hospital Muenster, Albert-Schweitzer-Campus 1, 48149 Muenster, Germany; 2 Institute for Immunology, University of Muenster, Roentgenstraße 21, 48149 Muenster, Germany; 3 Institute for Physiological Chemistry and Pathobiochemistry, University of Muenster, Waldeyerstraße 15, 48149 Muenster, Germany; 4 Cells-in-Motion Cluster of Excellence, University of Muenster, Waldeyerstraße 15, 48149 Muenster, Germany; 5 Division of Imaging Sciences & Biomedical Engineering, King’s College London, London, UK

## Abstract

Time-lapse MRI was implemented for dynamic non-invasive cell tracking of individual slowly moving intravascular immune cells. Repetitive MRI acquisition enabled dynamic observation of iron oxide nanoparticle (ION) labelled cells. Simulations of MRI contrast indicated that only cells moving slower than 1 µm/s were detectable. Time-lapse MRI of the brain was performed after either IONs or ION-labelled monocytes were injected intravenously into naïve and experimental autoimmune encephalomyelitis (EAE) bearing mice at a presymptomatic or symptomatic stage. EAE mice showed a reduced number of slow moving, i.e. patrolling cells before and after onset of symptoms as compared to naïve controls. This observation is consistent with the notion of altered cell dynamics, i.e. higher velocities of immune cells rolling along the endothelium in the inflamed condition. Thus, time-lapse MRI enables for assessing immune cell dynamics non-invasively in deep tissue and may serve as a tool for detection or monitoring of an inflammatory response.

## Introduction

Recent progress in the field of cell therapies^1,2^ and the increasing understanding of the complex interplay between different cell populations^3–5^ have created a demand for novel *in vivo* methods to longitudinally study the fate of specific cell populations or even individual cells. Optical techniques such as confocal or two-photon microscopy are well established for cell tracking, but require invasive procedures such as installation of cranial windows or skin-fold chambers^6,7^. This approach is therefore not suitable for all animal models, and has limited potential for clinical translation. Non-invasive cell tracking is possible by a number of different methods such as fluorescence or radionuclide imaging^8,9^ and different Magnetic Resonance Imaging (MRI) approaches using T2*w MRI of iron nanoparticle (ION)-labelled cells, ^19^F-MRI, or highly shifted proton MRI^10–12^. All of these methods have unique advantages which, however, are accompanied by drawbacks such as limited tissue penetration, instability of the marker, low spatial resolution, high background signal or limited sensitivity. With regards to potential clinical translation, T2*w MRI using ION-labelled cells offers the advantages of unlimited tissue penetration, stability of the marker substance, high spatial resolution, and additional morphological information^13–20^. However, due to the long image acquisition times, MRI and other non-invasive imaging methods could only acquire a static “snap shot” of labelled cells until recently. Although migration of cells has been detected by identifying cells at different locations at different time points, the actual movement remained concealed^17,21^. However, the direct observation of individual moving cells by MRI still seemed challenging until the concept of MRI time-lapse imaging was successfully implemented^18^. In this method, the established fluorescence microscopy time-lapse concept^6,7^, which collates sequentially acquired individual images into a movie that tracks migrating cells, was applied to MRI through repetitive acquisition of a series of static T2*w images. The time-lapse concept has recently been extended by performing real-time MRI acquisitions to visualize and assess the inflow and distribution of labelled cells in brain and spine in different animal models^22^. However, this approach did not aim at resolving single cells, but detected bulk signal of grafted cells from the vasculature directly after injection with a temporal resolution of two seconds. The detection of single monocytes was previously shown to be feasible with time frames of 20 minutes^18^. Multi-slice time-lapse acquisitions with whole-brain coverage provided movies tracking individual labelled monocytes in the vasculature of rat brain non-invasively. Yet, the strengths of such dynamic cell tracking has not been exploited in a clinical disease model^18^, and the temporal range of single cell motion that could be potentially resolved by time-lapse MRI was not addressed previously. The range of cellular velocities is of particular interest. Without any inflammatory stimulus, monocytes have been shown to patrol the endothelium at a velocity of approximately 0.2 µm/s, before being eventually dragged away in the blood stream with much higher speed^6,23^. Upon inflammatory stimuli, monocytes start rolling on the endothelium at approximately 40 µm/s and potentially extravasate into the surrounding tissue^6^.

Here, we aim to determine the velocity range that can be resolved with time-lapse MRI and to assess whether altered motion patterns of labelled leukocytes upon an immune response can be detected with this methodology. We use a murine model of experimental autoimmune encephalomyelitis (EAE)^24,25^ and compare it to healthy mice to assess whether time-lapse MRI is able to resolve different leukocyte motion patterns in the naïve and inflammatory state.

## Results

### Development of time-lapse MRI protocol

**Figure 1:**
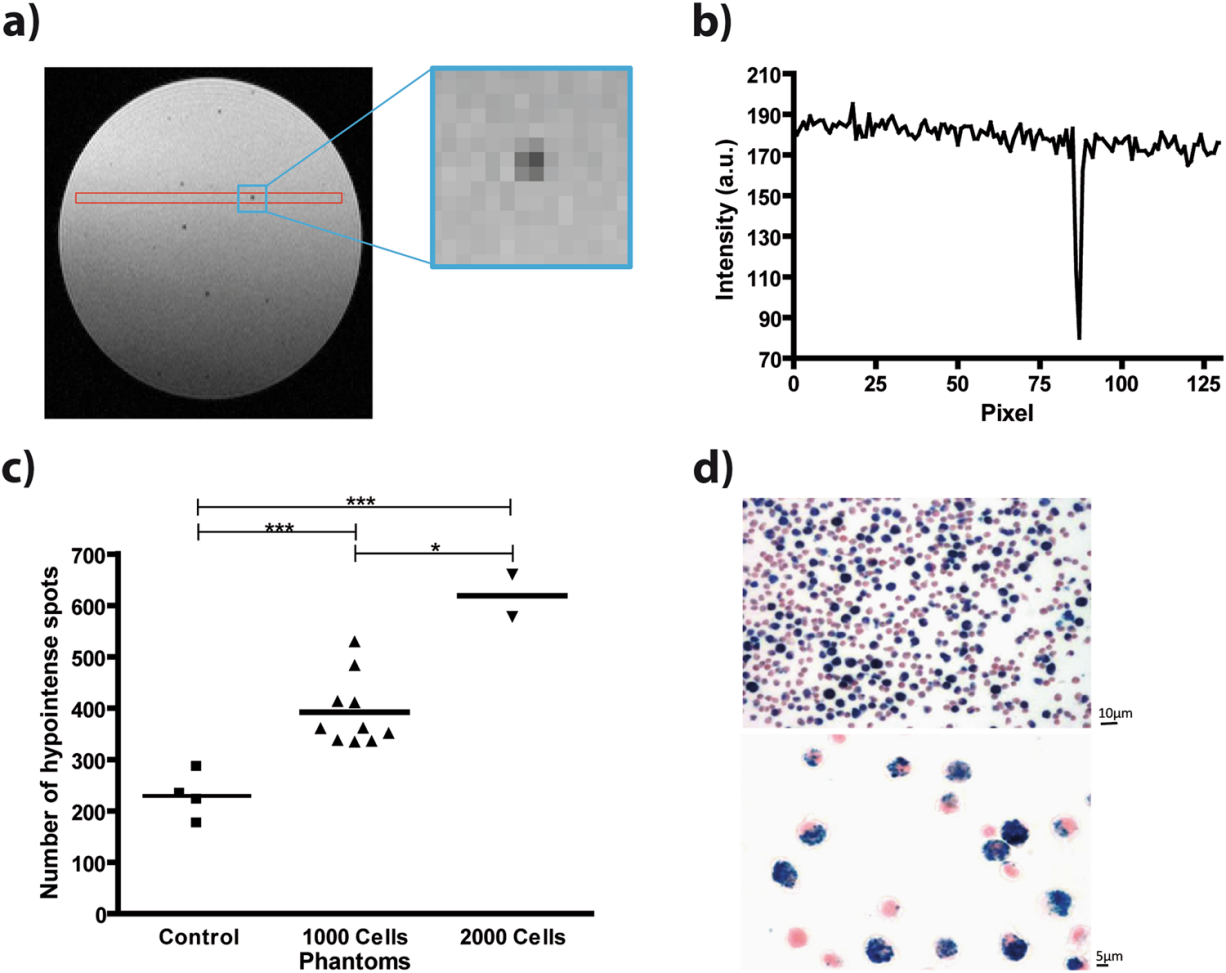
Time-lapse MRI is able to detect individual labelled monocytes. (**a**) Representative slice of an agarose gel phantom (1.5 ml tube) obtained with the time-lapse MRI protocol. Iron labelled HoxB8 monocytes can be identified as hypointense spots. Blue square indicates a representative zoom of one signal void, showing lowest signal in one central voxel, recovering over the two neighbouring voxels. (**b**) Representative profile plot (as indicated by red box in (**a**)) through the hypointense spot shows a maximum loss of signal intensity of about 70%. (**c**) Quantification of hypointense spots within the phantoms shows a significant increase in hypointense spots with increasing number of embedded labelled cells. The baseline amount of spots in control phantoms with no cells was attributed to air microbubbles (*p < 0.05, **p < 0.01 and ***p < 0.001). (**d**) Prussian blue staining of labelled cell suspension confirmed cell label and showed no free iron (100 µg Fe/ml; upper row: 20x magnification; lower row: 40x magnification).

A time-lapse MRI protocol with frame rate of 8 min 12 s was implemented to cover the whole mouse brain with a spatial resolution of 61 µm by 55 µm in 0.3 mm contiguous slices. To verify that this protocol was able to detect single labelled cells, measurements in agar gel phantoms with and without embedded ION-labelled monocytes were performed. The protocol provided images with a mean signal-noise ratio (SNR) of 35 ± 5. Inspecting the individual signal voids showed that signal was decreased in one central voxel by ~70%, slowly recovering over the two to three neighbouring voxels in all four directions (Fig. 1a,b). Quantitative analysis showed a significantly increased number of signal voids, depending on the number of ION-labelled cells embedded in the gel (Fig. 1c): an average of 230 ± 23 signal voids was measured in gel phantoms without embedded cells, which was attributed to microscopic air bubbles in the gel; 392 ± 21 signal voids for 1000 embedded labelled cells and, for two samples of 2000 embedded labelled cells, 578 and 660 signal voids were measured. Hence, 1000 cells resulted in roughly 200 additional signal voids. This was in agreement with the degree of ION cell labelling, as observed in Prussian blue stained cells (Fig. 1d). Microscopy further confirmed that no free iron particles were found in the cell suspension (Fig. 1d). The amount of iron per cell as measured by spectrophotometry (4.41 ± 2.19 pg/cell, n = 15) and T2-relaxometry (5.51 ± 2.64 pg/cell, n = 15) confirmed sufficient labelling for MRI detection.

### Simulations to define detectable velocity range of time-lapse MRI

**Figure 2:**
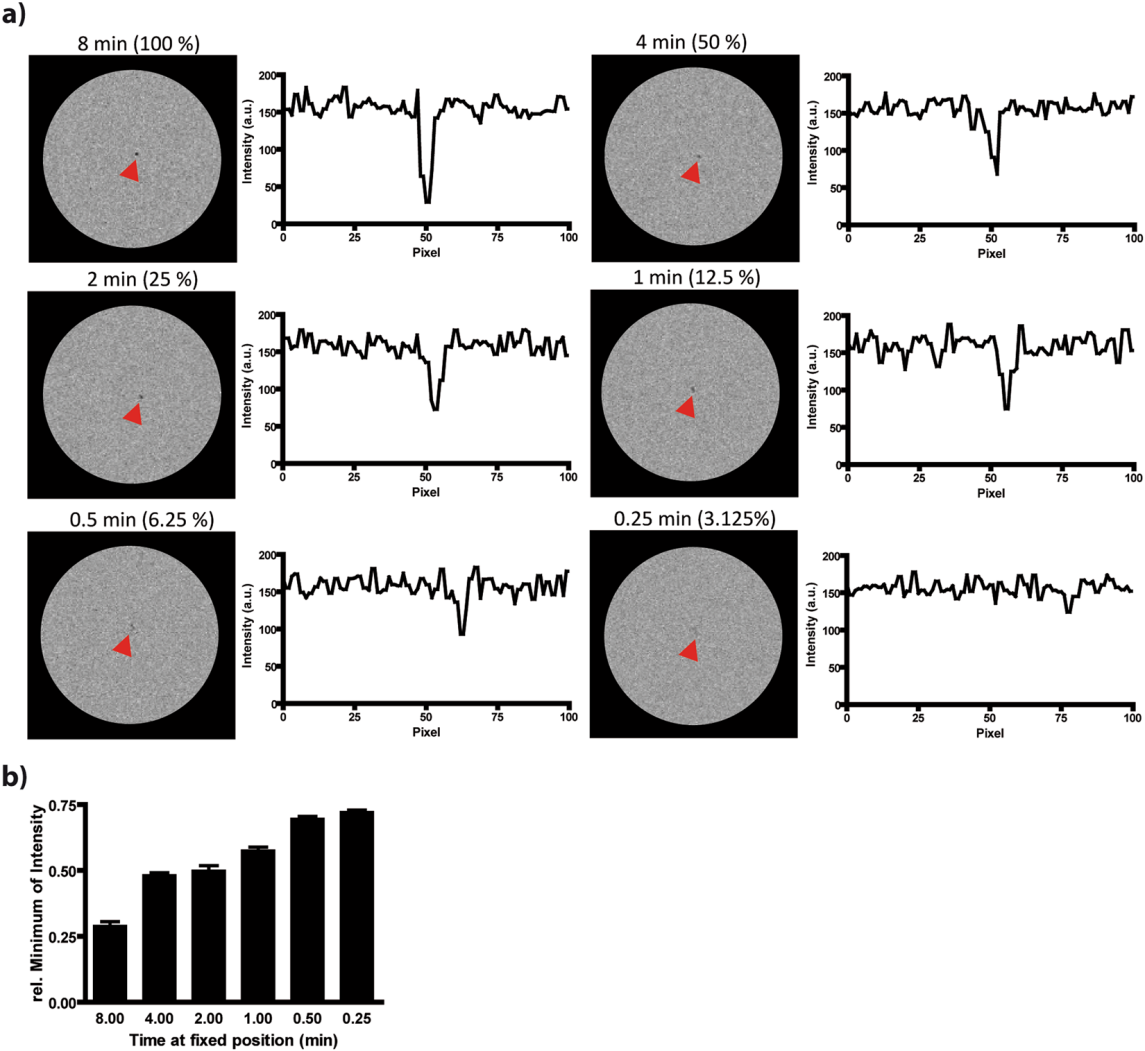
Simulations show that time-lapse MRI is able to detect moving cells. (**a**) Different motion velocities of a cell in a synthetic phantom were simulated by calculating images from synthetic k spaces. In the static case, the cell remained 8 min (100% of k space from one position) at a fixed position during the acquisition of one time frame. For moving cells, the resident time (percentage of k space from one position) was stepwise reduced. Cells could be observed even if k space was composed of 16 different positions (6.25% from one position). Profile plots show signal in horizontal line with maximum reduction. (**b**) Observed minimum signal intensity in the simulated images for different simulated motion velocities (n = 10 simulations each). Signal reduction decreased with higher simulated velocities.

In the next step, we assessed the ability of our time-lapse protocol to resolve cell motion. To define the range of velocities that could be captured by time-lapse MRI, we performed simulations in a synthetic phantom in silico, reproducing SNR and signal voids as observed in the measurements of static cells in the gel phantoms (Fig. 2). In the simulated images for the static case, a signal reduction in the central voxel to 29 ± 5% was observed (Fig. 2a). With increasing velocity, i.e. with higher numbers of motion steps during data sampling, the signal reduction decreased, while the labelled cells remained detectable in the two-dimensional images and line profiles (Fig. 2a,b). The simulations showed that contrast similar to the static situation was observed if cells remained at a fixed position for 50%, 25% and even 12.5% of the acquisition time. We therefore conclude that cells moving slower than the dimension of one pixel (61 × 55 µm) per minute, or approximately 1 µm/s are detectable with our time-lapse protocol.

### Time-lapse MRI after *in vivo* ION-labelling in healthy mice

**Figure 3:**
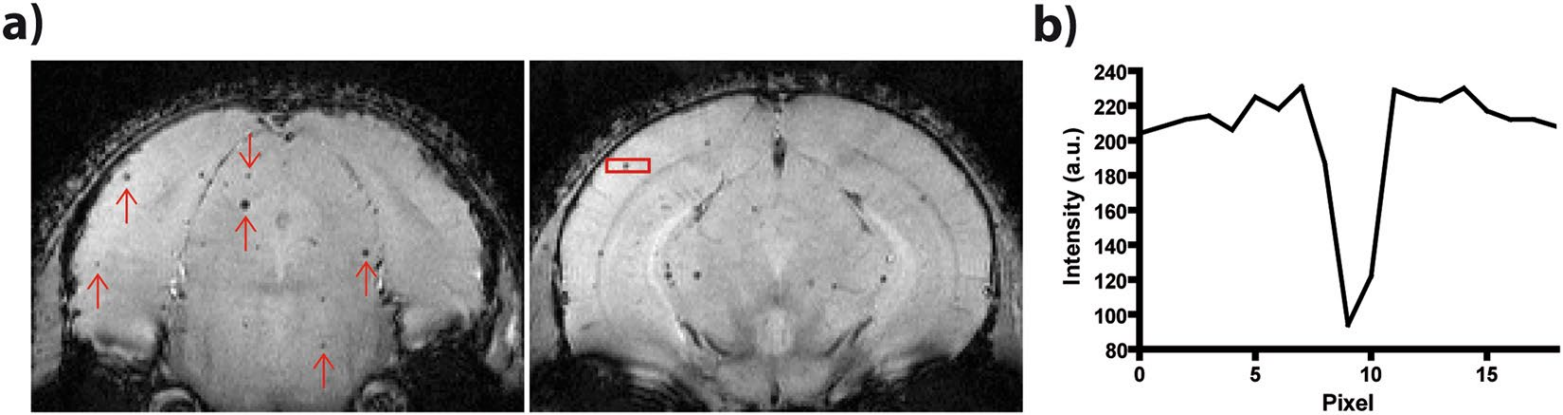
Time-lapse MRI is able to detect labelled innate immune cells *in vivo*. (**a**) Multiple hypointense spots (exemplarily marked with red arrows) were detected in the mouse brain after intravenous injection of ION, as shown in two representative slices of a naïve mouse brain. (**b**) Profile plot through one representative hypointense spots, as indicated by red box in (**a**), shows signal loss of about 70%.

**Figure 4:**
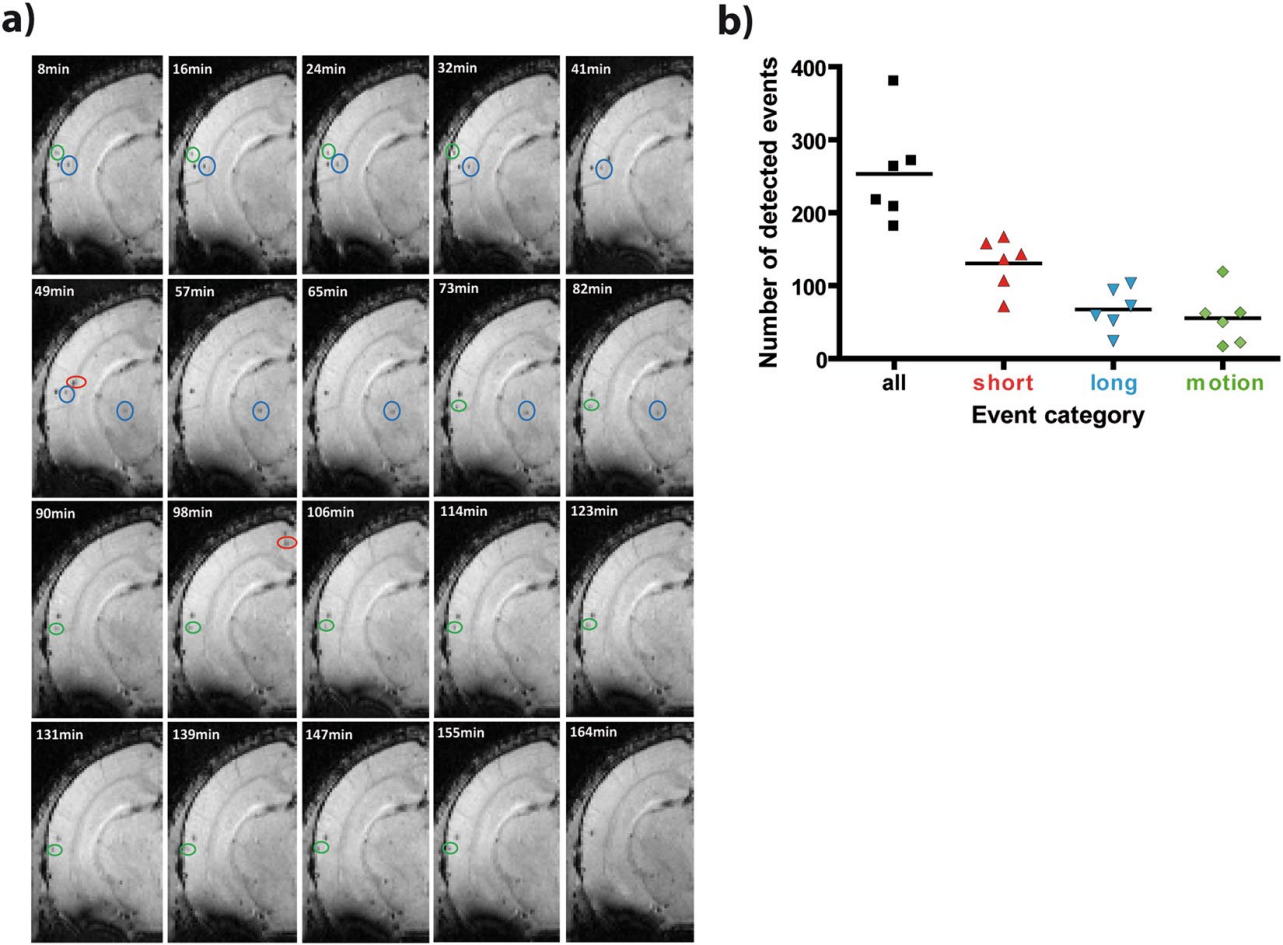
*In vivo* detected cells can be classified according to their velocities. (**a**) Representative example of the acquired 20 time frames of one slice from a naïve mouse brain reveals different motion velocities *in vivo*. Hypointense spots, representing labelled immune cells, were classified in three categories as motion (green circles), short (red circles) and long events (blue circles). (**b**) Quantification showed that substantial numbers of events from each category were observed in each of the naïve mice (n = 6).

Next, we applied our time-lapse protocol to naïve mice *in vivo*, yielding average SNR values of 33 ± 8 in cortical and 31 ± 4 in subcortical regions of the brain. For the purpose of cell tracking, mononuclear phagocytes were labelled by i.v. injection of ION^18,26,27^. Varying numbers of hypointense spots were observed in all slices through the brain (Fig. 3a). The hypointensities typically extended over three to five voxels along each direction and showed signal reductions of up to about 70% in the central voxel (Fig. 3b). Due to the highly similar contrast pattern as compared to the phantom measurements, we conclude that the hypointensities are caused by labelled innate immune cells. Acquisition of multiple time frames allowed collation of all images into time-lapse movies, which revealed the 3D dynamics of labelled cells moving through the brain (Supplemental Videos 1 and 2). To characterise and quantify the dynamics of the observed labelled cells, we counted the hypointense spots as “events”. These were arbitrarily subcategorised in short (detected in one or two consecutive time frames), long (three or more consecutive time frames) or motion events (observed motion in-slice or to a consecutive slice in three or more consecutive time frames). In naïve mice, a total of 253 ± 29 (n = 6) events were detected, most (52%) of which were short events (131 ± 14); 26% were categorized as long stationary (67 ± 12) and 22% as motion (56 ± 15) events (Fig. 4a,b).

To assess the potential impact of artifacts detected as false positives, we performed time-lapse MRI with the identical protocol in naïve healthy mice (without ION injection, n = 3). Analysis showed a number of 6 ± 1 spherical or single voxel hypointensities, which potentially could have been mistaken as events. All these events were observed only in one time frame, i.e. categorized as short events. No motion or long events were observed in this baseline control.

### Time-lapse MRI after *in vivo* ION-labelling in EAE mice

**Figure 5:**
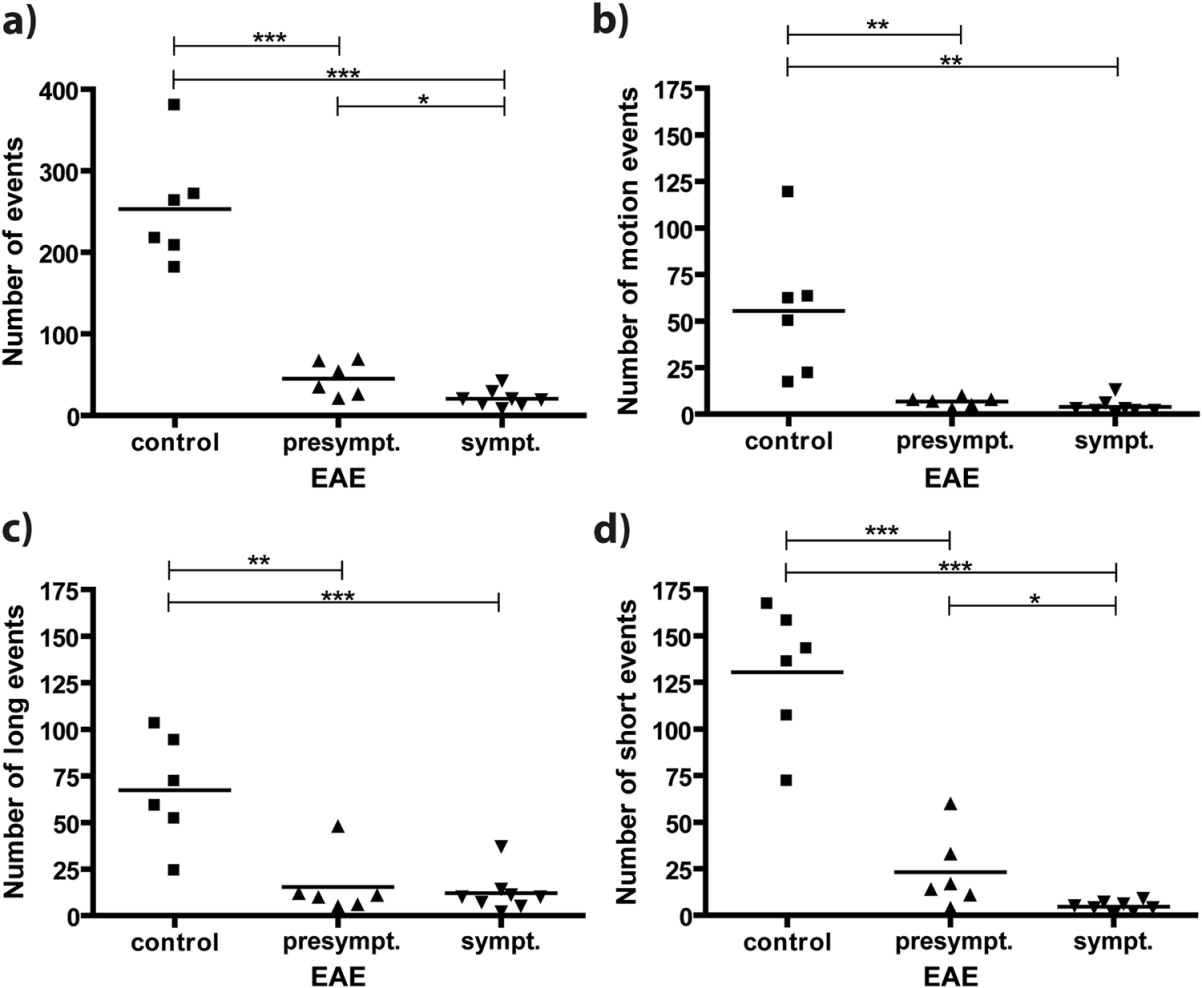
Detection of EAE upon direct i.v. injection of ION. (**a**) Overall events (number of counted hypointense spots) were significantly higher in naïve as compared to EAE mice, both before (EAE score 0) and after onset of symptoms (EAE score 1–3). The difference between presymptomatic and symptomatic mice was significant. Differences were observed for all subcategories: (**b)** motion (**c)** long and (**d)** short events (*p < 0.05, **p < 0.01 and ***p < 0.001).

Following the successful assessment of our time-lapse sequence in phantom scans and naïve mice, we applied our protocol to explore the dynamics of immune cells after an immune response had been triggered, using EAE mice as a model of neuroinflammation. EAE mice were grouped into presymptomatic (score 0, n = 6) and symptomatic animals (score 1 to 3, n = 8), and subjected to i.v. injection of ION. Similar to naïve mice, we were able to observe both static and dynamic hypointense spots, most likely representing labelled immune cells (Supplemental Video 2). However, the total number of detected events was significantly lower in EAE mice as compared to naïve controls, both at symptomatic (21 ± 4) and at presymptomatic (45 ± 9) stages of the disease (Fig. 5a). This significant difference between naïve and EAE mice was observed in all three subcategories: The number of cells moving slower than the estimated detection threshold of 1 µm/s dropped from 56 ± 15 (control mice) to 7 ± 1 (presymptomatic EAE) and 4 ± 1 (symptomatic EAE), long events from 67 ± 12 to 15 ± 7 and 12 ± 4 and short events from 131 ± 14 to 23 ± 8 and 5 ± 1, respectively (Fig. 5b–d). These numbers represent changes in the percentage of each subcategory with respect to the overall number of events. For short events, a decrease to 23% from 52% was observed, in symptomatic EAE mice compared to healthy mice, and for long events an increase to 57% from 26%, respectively (Table 1). The method further revealed a significant difference in the number of observed overall and short events in EAE mice after onset of clinical symptoms compared to presymptomatic mice (Fig. 5a,d), suggesting that the majority of the cells were moving outside of the detection limits.

**Table 1:** Percentage of events per category (short, long, motion) compared to overall number of events in the brain for control, presymptomatic and symptomatic EAE mice.

	control (of 253 total events)	presymptomatic (of 45 total events)	symptomatic (of 21 total events)
short events	52%	51%	23%
long events	26%	33%	57%
motion events	22%	15%	19%

### Time-lapse MRI with *in vitro* ION-labelled ER-HoxB8 monocytes in EAE

Next, we assessed whether our time-lapse protocol was suitable for *in vivo* detection of an *in vitro* labelled cell line. For this purpose ION labelled monocytes from ER-HoxB8 progenitor cells^28^ were injected i.v. into naïve (n = 3) and EAE mice (presymptomatic, n = 11 and symptomatic, n = 16).

**Figure 6:**
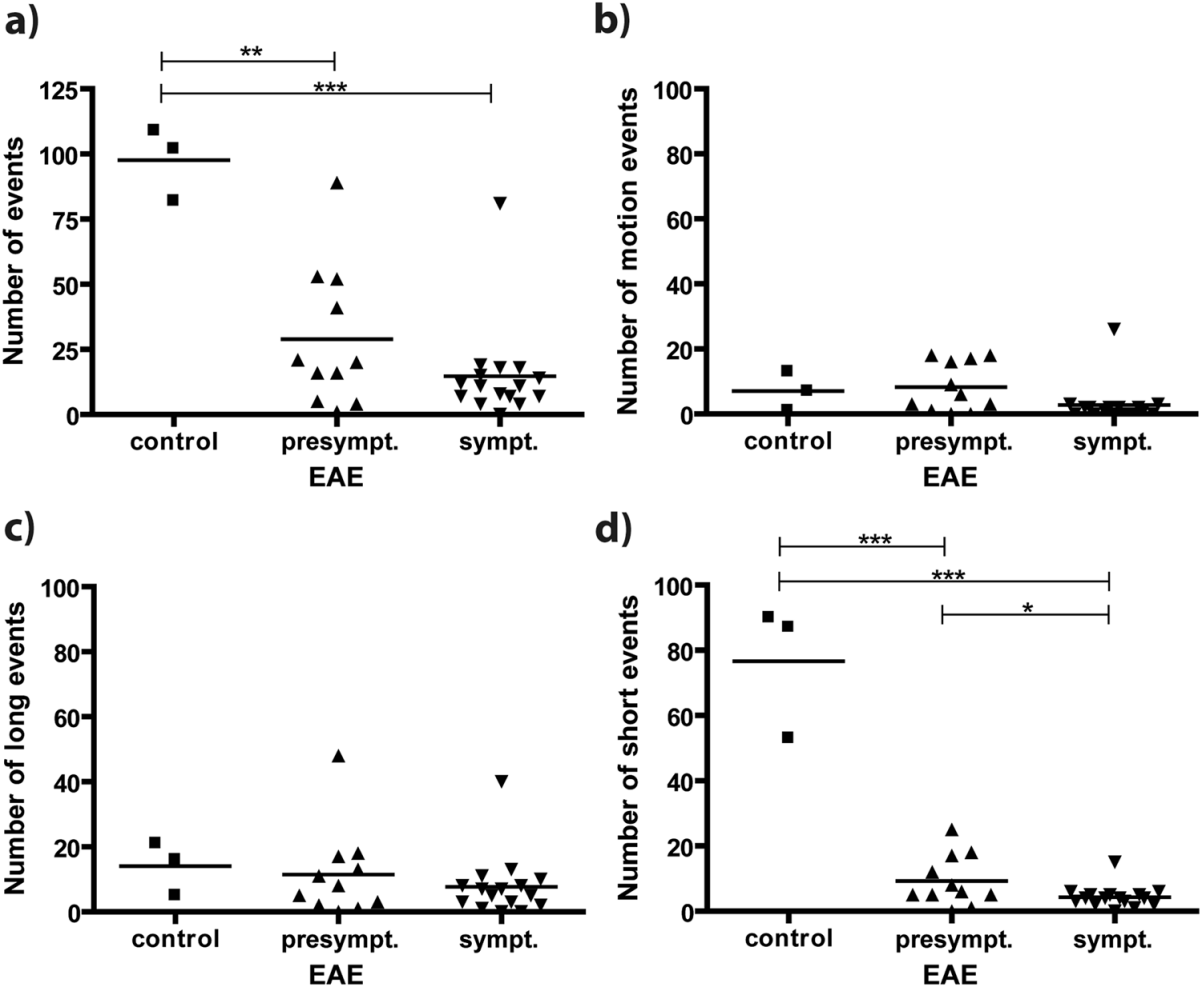
Detection of EAE upon i.v. injection of ION-labelled ER-HoxB8 monocytes. (**a**) Overall events (number of counted hypointense spots) were significantly higher in naïve as compared to EAE mice, both before (EAE score 0) and after onset of symptoms (EAE score 1–3). No significant difference was observed between presymptomatic and symptomatic mice. No significant differences were observed in the subcategories (**b**) motion (**c)** long events. (**d**) For short events, significant differences were observed between naïve and EAE mice, as well as between presymptomatic and symptomatic mice (*p < 0.05, **p < 0.01 and ***p < 0.001).

In naïve mice, hypointense spots representing labelled cells were detectable. However, compared to direct i.v. ION injection, mice injected with labelled ER-HoxB8 monocytes showed a significantly lower number of overall detected events, 98 ± 8 (Fig. 6a) versus 253 ± 29 for *in vivo* labelling (Fig. 4b). While still a substantial number of short events was detected (77 ± 12, Fig. 6d compared to 131 ± 14 for *in vivo* labelling, Fig. 4b), there were hardly any motion (7 ± 3, Fig. 6b) or long (14 ± 5, Fig. 6c) events detected. These two subcategories were reduced compared to the *in vivo* labelling approach (67 ± 12 and 56 ± 15, respectively, Fig. 4b).

**Figure 7:**
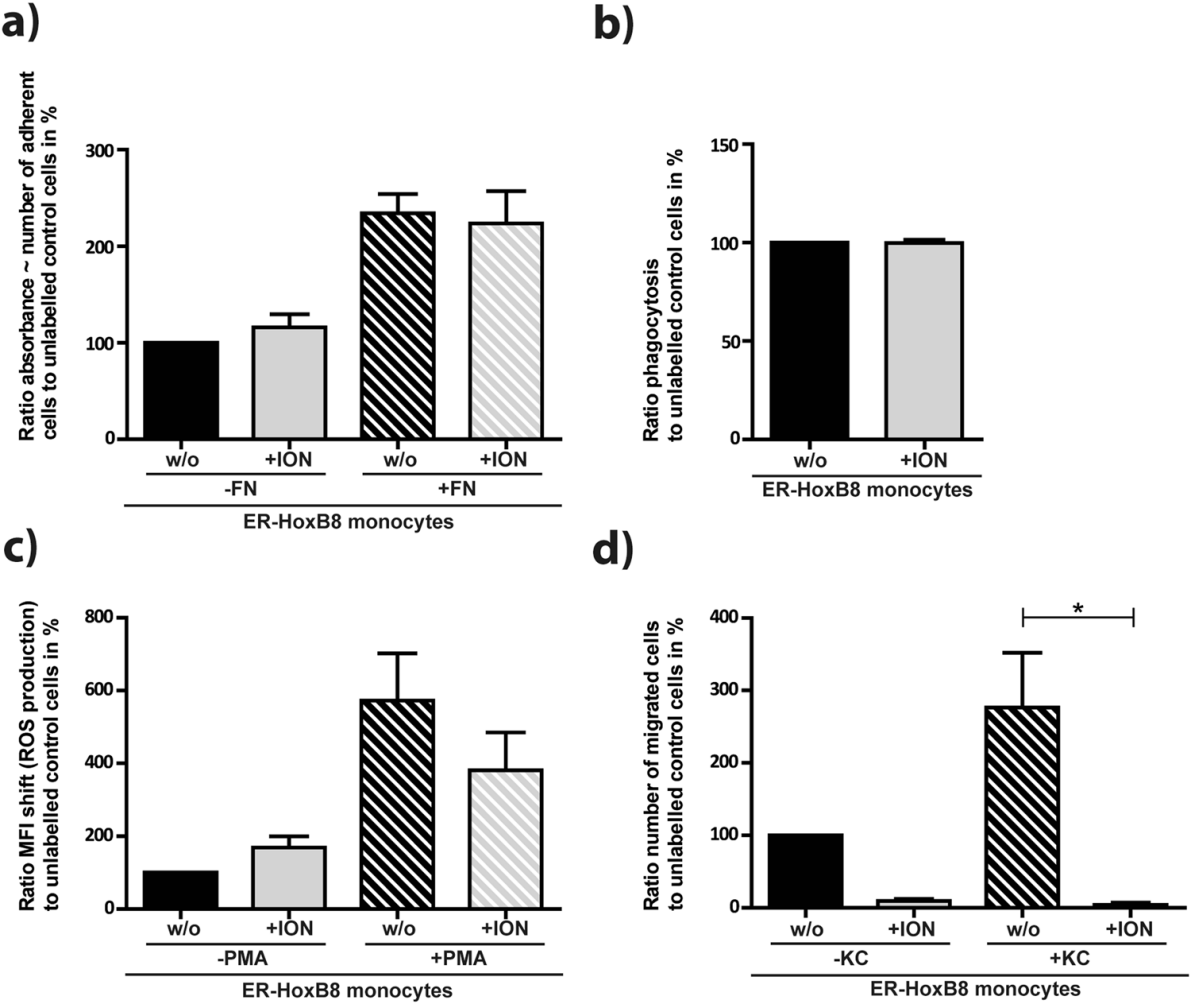
ION-labelling compromises migratory capacity of ER-HoxB8 monocytes. Functionality assays of ION-labelled ER-HoxB8 monocytes showed that (**a)** adhesion (**b**) phagocytic performance and (**c**) ROS production were not affected by ION-labelling while (**d**) migratory capacity was significantly reduced after labelling (FN = Fibronectin, ION = ION labelling, KC = CXCL1, PMA = Phorbol-12-myristat-13-acetat, w/o = without, *p < 0.05).

As these results suggest altered motility of the *in vitro* compared to *in vivo* labelled cells, we performed functionality assays with the *in vitro* labelled ER-HoxB8 monocytes. The key functions of adhesion, phagocytosis and reactive oxygen species (ROS) production remained unchanged compared to unlabelled control cells (Fig. 7a,b,c). Hence, the labelled cells were still viable in mice and could be detected by time-lapse MRI. The migration capacity, however, was significantly decreased after ION-labelling, as reflected in a 90% reduction of spontaneous migration rate and 98% reduction of chemokine stimulated migration rate as compared to unlabelled control cells (Fig. 7d). Interestingly, time-lapse MRI was capable of resolving this restricted motility by analysing the different types of events observed.

Similar to the *in vivo* labelling approach, for *in vitro* labelled ER-HoxB8 monocytes the number of detected overall events was significantly reduced in both presymptomatic (n = 11) and symptomatic (n = 16) EAE mice (Fig. 6a). In particular, time-lapse MRI was capable of detecting significantly altered immune cell dynamics in presymptomatic (29 ± 8) as compared to naïve mice (98 ± 8, Fig. 6a). However, the number of long and motion events was low in both EAE groups (Fig. 6b,c) and the loss of total events in EAE mice was mainly due to a significant reduction in short events (9 ± 2 for presymptomatic and 4 ± 1 for symptomatic EAE vs. 77 ± 12 for naïve mice, Fig. 6d).

We conclude that our *in vitro* labelling results are in accordance with our *in vivo* labelling results, showing enhanced cell motility after an inflammatory response has been triggered and thereby reduced detectable motion signals by time-lapse MRI.

## Discussion

Although there have been various studies assessing MRI cell tracking, either by acquiring a snapshot at different locations and time points or by addressing cell distribution^11,17,21,22^, the underlying cell movement remained concealed. The original implementation of time-lapse MRI was a technical milestone, for the first time capturing intravascular motion of individual monocytes non-invasively^18^. Our data suggest that time-lapse MRI can distinguish between different immune cell dynamics in healthy versus diseased states. This conclusion relies on three preconditions: (1) relevant immune cells were labelled and detected, (2) the velocity range resolved by time-lapse MRI has been estimated correctly, and (3) the EAE model provided an appropriate inflammatory stimulus that causes a different motion pattern of monocytes compared to healthy mice.

Previous studies have shown that MRI is able to detect labelled immune cells^18,27,29^. Our experimental data showed spatial and temporal patterns comparable to the first publication of time-lapse MRI, where hypointensities were attributed to individual labelled immune cells in the brain^18^. We therefore conclude that the hypointensities observed in our images originate from individual labelled immune cells. This is further corroborated by the high similarity in appearance of the hypointensities compared to our observations of *in vitro* labelled monocytes and to observations of single labelled cells by others^13–20^. Previous histological analyses further revealed that most of hypointensities display intravascular cells^4,18^. It is important to note that the temporal resolution of time-lapse MRI as presented here is not high enough to follow cells travelling with the blood stream^22^, but rather detects labelled cells during their slow active movement along the endothelium and potentially after extravasation into the adjacent tissue.

Although a contribution of free iron to the observed contrast cannot be fully excluded, we assume that this is negligible for the following reasons. IONs moving freely in the blood stream are expected to have velocities far beyond 1 µm/s and are thus too fast to be detectable by time-lapse MRI. Further, the small particle size of the IONs renders detection of individual particles with MRI virtually impossible. Finally, the number of ION in the blood is expected to have substantially decreased 24 h after injection. Similarly, a significant contribution of not iron-related artefacts to the number of counted events is highly unlikely. Control measurements in naïve mice showed that the number of potential false positives was substantially lower than the number of event observed after ION injection and may therefore be considered as negligible.

The range of velocities resolved by our time-lapse MRI protocol was derived from simulations using synthetic phantoms that reproduced experimentally observed contrast. The simulations showed that contrast of moving cells was critically determined by the time cells remained within the dimensions of one voxel. Therefore, our estimate of velocities < 1 µm/s as detection limit of our time lapse protocol appears stable against parameter variations. Even if our calculated velocity range deviated from the experimental situation by a factor of ten, time-lapse MRI still resolves the relevant difference of 0.2 µm/s for healthy versus 40 µm/s for diseased state.

The presence of patrolling monocytes (at a mean velocity of 0.2 µm/s) in the vasculature of healthy animals has been shown previously^6,23^. This velocity range is accessible by time-lapse MRI, explaining the large number of detected cells in healthy mice in our measurements. While in the healthy state monocytes ‘patrol’ the vasculature screening for pathogens and other potential stimuli along the resting endothelium, monocyte abandon their patrolling behavior upon an inflammatory stimulus and start rolling (at a mean of 40 µm/s), with the aim to leave the circulation into diseased tissue^6^. Therefore, the faster moving cells are no longer detectable resulting in a reduction in the number of observed hypointense spots. There is ample evidence that EAE provides an inflammatory stimulus inducing such rolling of leukocytes^4,30,31^. A transition from patrolling to rolling cells is reflected by our data, showing a pronounced decrease especially of the short event subcategory in symptomatic EAE mice compared to healthy mice. The corresponding relative increase of the long event subcategory still represents a strong decrease in absolute numbers, and may be due to very few extravasating cells. While EAE models generally induce endothelial transmigration of monocytes at focal lesions^24^, the myelin oligodendrocyte glycoprotein (MOG_35–55_)-induced model used in this study is known to induce only low lesion loads in the cerebrum, compared to the spinal cord, or compared to other EAE models^27,32^. In agreement with this notion, our data did not show massive monocyte infiltration into brain tissue in EAE mice. However, the systemic effect of the inflammatory stimulus was detected as a significantly reduced number of patrolling monocytes, compared to healthy controls. In EAE mice, monocytes simply moved too fast for detection by the presented time-lapse MRI approach. Although not limited to the brain, one has to acknowledge that time-lapse MRI of other regions of interest might be challenging due to increasing motion artifacts.

While the approach of *in vivo* labelling of monocytes and macrophages with ION^18,33,34^ may be useful for translation into clinical diagnostics, studying and tracking specific cells is a key element of basic research. This requires *in vitro* labelling of isolated or cultivated cell lines, which are subsequently injected into the animal. We used ER-HoxB8 monocytes that were efficiently labelled as confirmed by Prussian blue staining, T2-relaxometry and spectrophotometry. Iron load was found to be sufficient for MRI detection, although it was lower than described for human monocytes^35^, which might be due to altered internalization properties of the applied cell line. Our time-lapse protocol was able to detect and track labelled ER-HoxB8 cells *in vivo*, though the number of observed hypointensities was far lower than after direct i.v. contrast agent injection for both healthy and EAE mice. This decrease may be caused by *in vivo* elimination of foreign cells by the host immune system or by homing to the liver. Further, a reduced percentage of motion events observed for grafted *ex vivo*-labelled ER-HoxB8 cells (7%) as compared to *in vivo*-labelled endogenous cells (26%) hints at altered cell dynamics of these two populations and may contribute to the reduced number of events observed with *ex vivo* prelabelled cells. Functional assays of labelled ER-HoxB8 cells confirmed functional impairment of migratory capacity maybe due to iron overload or higher iron toxicity of this cell line, while vitality and other key functions were intact. Interestingly time-lapse MRI was able to depict this restricted cell function.

In conclusion, we showed that time-lapse MRI is capable of resolving immune cell dynamics at a single-cell level *in vivo* non-invasively. Both *in vitro* and *in vivo* labelled cells can be tracked dynamically within a sensitive temporal window. In an EAE mouse model, alterations in cell dynamics were detected before the onset of clinical symptoms, suggesting that time-lapse MRI may provide a versatile tool for depicting the onset of immune response.

## Methods

### Animals

Animal husbandry and experimental manipulation were carried out according to local animal welfare guidelines and were approved by the LANUV, Recklinghausen, Germany (ID: 84-02.04.2011.A087). Female C57BL/6 mice (n = 50) were obtained from Charles River Laboratories (Sulzfeld, Germany) and housed under a 12 h light–dark cycle and provided with food and water ad libitum. Experimental autoimmune encephalomyelitis (EAE) was induced (n = 41) using myelin oligodendrocyte glycoprotein (MOG_35–55_) as previously described^24,25,36^. Mice were monitored daily for disease symptoms, and scored on a scale from 0 to 5 (see Table 2). Disease peaked between days 14 and 18 after immunization^24^.

**Table 2:** EAE Score according to disease symptoms of the mice.

EAE Score	Clinical Symptoms
0	no clinical symptoms
1	limp tail
2	hind limb weakness
3	severe hind limb weakness
4	hind quarter paralysis
5	immobilization or death

### Contrast agents and cell labelling

For *in vivo* labelling, Ferucarbotran (Resovist®, Bayer AG), an approved standard ION (50–100 nm), was used as MRI contrast agent. 24 h prior to MRI, 1.3 ml per kg/body weight was injected i.v. via the tail vein into naïve, presymptomatic (day 10 post EAE immunization, score 0) or symptomatic (day 13–17 post EAE immunization, score 1–3) mice and labelled with Resovist®.

### Labelling of ER-Hoxb8 cells

For *in vitro* labelling, monocytes from ER-HoxB8 progenitor cells were cultured and differentiated as previously described^28^. ER-Hoxb8 cells were transferred to 6-well plates at a concentration of 1 × 10^6^ cells/ml RPMI1640 medium and 100 µg Fe/ml (Resovist®). After 4 h incubation time, cell supernatant was discarded and attached cells were washed with warm (37 °C) PBS four times and treated with 0.5% Trypsin 0.2% EDTA for 3 min at 37 °C. Detached cells were collected, centrifuged (282 g, 1300 rpm, 7 min). 4–6 × 10^6^ labelled cells were injected intravenously into either (pre-)symptomatic EAE or naïve control mice.

Prussian blue staining, T2-relaxometry and spectrophotometry for confirmation of ION labelling of ER-HoxB8 monocytes was performed. Prussian blue staining followed standard protocols. For T2-relaxometry 0.1 × 10^6^ labelled ER-HoxB8 were embedded in 1 ml of 1% agarose phantom (n = 15, independent), followed by MRI at 9.4 T (Bruker BioSpec). For T2-relaxometry a multi spin multi echo (MSME) sequence with following sequence parameters was used: TR: 2500 ms, TE: 5.93 ms, echoes: 30, refocusing FA: 180°, averages: 2, repetitions: 1, matrix: 128 × 128, scan time: 8 min. Iron load per cell was determined via calibration with a serial dilution of ION. Additionally, spectrophotometry was performed as described previously^37^. Briefly, 0.5 × 10^6^ labelled ER-HoxB8 monocytes were lysed in 1.5 ml of 10% SDS (n = 15, independent) and measured via spectrophotometry (Hitachi U-3010) at A_370 nm_ (for iron oxide quantification) and A_750 nm_ (for correction of turbidity of cellular products). Iron oxide content per cell was quantified via calibration with a serial dilution of ION.

### Functional assays of ION labelled ER-HoxB8 cells

Functionality of labelled ER-HoxB8 cells was analysed by *in vitro* assays for adhesion, transwell-migration, reactive oxygen species production and phagocytosis.

#### Transmigration Assay

A transwell migration assay on the basis of a two chamber model was performed. Optionally, LTB4 as chemotactic trigger was added in a concentration of 12 nM. Cells in a concentration of 1 × 10^6^ cell/100 µl medium were put on top of the filter membrane (5 µm pore size, Corning®Transwell®, Sigma Aldrich, St. Luis, USA). Cells were allowed to transmigrate for 1 h at 37 °C. Number of migrated cells was determined by flow cytometry analysis (FACS Calibur, CellQuestPro software, Becton-Dickson, Heidelberg, Germany).

#### Adhesion assay

Wells of a 24-well plate were either coated with fibronectin (25 µg/ml, Roche, Penzberg, Germany) or left untreated. Cells were seeded in a concentration of 5 × 10^5^ cells in each well and allowed to adhere for 2 h, 37 °C. Adherent cells were fixed with 2% glutaraldyhde (10 min, RT) and stained with 0.5% crystal violet (in 200 mM Boric acid, pH 8). Cells were lysed with acetic acid and transferred to a 96-well plate. Adhesion was determined as absorbance at 560 nm.

#### Phagocytosis assays

Cells were diluted to 5 × 10^5^ cells/500 µl and incubated for 4 h, 37 °C with 5 × 10^6^ fluorescent labelled beads/500 µl (FluoSpheres® Fluorescent Microspheres, 1 µm, 530/30 nm Thermo Fisher Scientific, Waltham, USA). Cells were analysed for phagocytosed fluorescent beads by flow cytometry, FL-1H (FACS Calibur, CellQuestPro software, Becton-Dickson, Heidelberg, Germany).

#### ROS production assay

Cells were diluted to 5 × 10^5^ cells/500 µl and stimulated with 100 nM PMA (Phorbol 12-myristate 13-acetate, Abcam, Cambridge, USA) or left untreated. Cells were incubated for 45 min, 37 °C and 15 µM dihydrorhodamine 123 was applied and incubated for another 15 min, 37 °C. Cells were analysed by flow cytometry, FL-1H (FACS Calibur).

### MRI

MRI was performed on a 9.4 T Biospec (Bruker Biospin, Ettlingen, Germany) using a cryogenic probe. For time-lapse MRI, as a compromise between highest spatial resolution to resolve individual cells and high temporal resolution to efficiently resolve dynamics, we developed a T2* gradient echo sequence with the following scan parameters: TR: 649 ms, TE: 8.0 ms, FA: 60°, averages: 4, matrix: 180 × 256, in-plane resolution: 61 × 55 µm^2^, 38 contiguous slices, slice thickness: 300 µm, scan time: 8 min 12 s (single time frame) resulting in 2 h 44 min for 20 repetitions.

Phantom experiments were performed with agarose gel phantoms, containing either no (n = 4), 1000 (n = 10) or 2000 (n = 2) ION-labelled ER-HoxB8 monocytes. Cells were mixed gently in 1 ml of 1% agarose in 1.5 ml tubes and immediately put on ice to harden the agarose with embedded labelled cells.

For *in vivo* studies the MRI time-lapse protocol was used for EAE and naïve control mice with 20 time frames. Either direct i.v. ION injection for *in vivo* labelling of innate immune cells (n = 8 for symptomatic, n = 6 for presymptomatic EAE, n = 6 for naïve control mice) or i.v. injection of *in vitro* ION-labelled ER-HoxB8 monocytes (n = 16 for symptomatic, n = 11 for presymptomatic EAE, n = 3 for naïve control mice) was evaluated. MRI of the brain was performed 24 h after ION-labelled cell injection. Additionally, three time-lapse MRI scans were performed as baseline control in naïve healthy mice without ION injection (n = 3). Mice were anesthetized with 1.5% isoflurane in 1 L per minute of oxygen and compressed air (20:80) under continuous respiratory and temperature monitoring. To avoid body cooling, mice were kept at physiologic temperature by a specifically designed animal heating device. Pronounced changes in body temperature or breathing frequency were stop criteria for the measurements. Data was discarded and no time-lapse MRI data analysis was performed under such unstable conditions.

### Analysis of MRI data

MRI data were processed using Matlab (The Mathworks, Inc., Natick, MA) and ImageJ (Version 1.50b, Wayne Rasband, National Institute of Health, USA). Correction of residual motion between different time frames was performed using SPM12 (Functional Imaging Laboratory, Wellcome Trust Centre for Neuroimaging, UK London). Signal to noise ratio (SNR) was defined as mean image intensity in regions of interest divided by standard deviation of the noise x 0.655, and calculated from n = 3 measurements each in independent phantoms or mice.

Detected hypointense spots (events) were manually counted and matched in all acquired slices and time frames of the brain. Subsequently, events were further subcategorised in short (detected in one or two consecutive time frames), long (three or more consecutive time frames) or motion events (three or more consecutive time frames and observed motion in-slice or to a consecutive slice).

### Simulations

To address the question of the velocity range of cell motion that can be resolved by time-lapse MRI, Matlab simulations were performed to assess the expected image contrast of labelled cells that are moving during data acquisition. For this purpose, a synthetic phantom of 256 by 256 voxels with intensity 1 was used. A synthetic labelled cell was added at varying positions, represented by three by three voxels of reduced intensity (0.3 for the central voxel, 0.5 for the four directly adjacent voxels, and 0.7 for the four diagonally neighbouring voxels), reproducing experimentally observed signal voids. Gaussian white noise was added to the phantom, using the function *imnoise* in Matlab, to achieve a standard deviation in the calculated images of 6.3%. This higher noise level as compared to the experimental images was chosen to partly account for the absence of potentially confounding structures and experimental artefacts in the simulations. Motion of the synthetic cell was simulated, moving the cell diagonally across the phantom by stepwise increasing both horizontal and vertical voxel position. For each position of the synthetic cell, the phantom was Fourier transformed to create a set of synthetic position-specific k space data. Finally, images for different simulated motion velocities were obtained by Fourier transforming reassembled synthetic velocity-specific k space data, composed of different fractions of the position-specific k space data. For zero velocity, the velocity-specific k space was identical to the position dependent k space. To simulate slow motion of only one voxel during image acquisition, the first half of velocity-specific k space (i.e. 128 lines) was filled with position-specific k space lines for one position, and the second half with position-specific k space lines for the adjacent position. To simulate motion over four voxels during image acquisition, the first quarter of velocity-specific k space (i.e. 64 lines) was filled with position-specific k space lines for one position, the second quarter with position-specific k space lines for the adjacent position, and so on. Higher motion velocities were simulated by filling smaller fractions (i.e. 32 lines, 16 lines, 8 lines) of the velocity-specific k space, before moving to the next position-specific k space. For analysis of the simulated images, all simulations were performed ten times in independent runs, and images were scaled to mean image intensity.

### Statistical analysis

Statistical analyses were performed with GraphPad Prism version 4.0 for windows (GraphPad Software, San Diego, USA). Results shown are means and standard error of the mean. P-values < 0.05, obtained with an unpaired two-sided Students t-test, were considered statistically significant.

## Electronic supplementary material

Legends Supplementary Videos

Supplementary Video 1

Supplementary Video 2

Supplementary Video 3
