# Supplementary material for: Temporal window for detection of inflammatory disease using dynamic cell tracking with time-lapse MRI
Source: Sci Rep. 2018 Jun 22;8:9563. doi: 10.1038/s41598-018-27879-z (PMC6015069; doi:10.1038/s41598-018-27879-z)
Supplement: Supplementary file 1 — Legends Supplementary Videos [file 41598_2018_27879_MOESM1_ESM.docx]

**Temporal window for detection of inflammatory disease using dynamic cell tracking with time-lapse MRI**

**Supplementary Info**

Max Masthoff (MD)^1^, Sandra Gran (PhD)^2^, Xueli Zhang (PhD)^3,4^, Lydia Wachsmuth (PhD)^1^, Michael Bietenbeck (MSc)^1^, Anne Helfen (MD)^1^, Walter Heindel (MD)^1^, Lydia Sorokin (PhD)^3,4^, Johannes Roth (PhD)^2,4^, Michel Eisenblätter (MD, PhD)^1^, Moritz Wildgruber (MD, PhD)^1,4^, Cornelius Faber (PhD)^1,4*^

1. Translational Research Imaging Center, Department of Clinical Radiology, University Hospital Muenster, Albert-Schweitzer-Campus 1, 48149 Muenster, Germany

2. Institute for Immunology, University of Muenster, Roentgenstraße 21, 48149 Muenster, Germany

3. Institute for Physiological Chemistry and Pathobiochemistry, University of Muenster, Waldeyerstraße 15, 48149 Muenster, Germany

4. Cells-in-Motion Cluster of Excellence, University of Muenster, Waldeyerstraße 15, 48149 Muenster, Germany

*Correspondence should be addressed to CF

email: faberc@uni-muenster.de

Tel: +49 (0) 251 83 57608, Fax: +49 (0) 251 83 52067

**Legends to supplemental videos**

**Supplemental video 1**

The video shows the collation of all 20 time frames of one brain slice 24 h after i.v. ION injection in a naïve mouse. Single time frame acquisition time was 8 min 12 s. Detected hypointense spots (events) representing labelled immune cells could be observed dynamically over time and were subcategorised in short (detected in one or two consecutive time frames), long (three or more consecutive time frames) or motion events (three or more consecutive time frames and observed motion in-slice or to a consecutive slice).

**Supplemental video 2**

Time-lapse movie with all twenty time frames of exemplary 20 slices (out of 38) of a naïve mouse brain 24 h after i.v. ION injection. The video reveals the 3D dynamics of labelled cells moving through the brain.

**Supplemental video 3**

The video shows an observation (overall for roughly 130 min) of a dynamic immune cell (green rectangle) in one brain slice of an EAE diseased mouse. Overall, detected hypointense spots (events) were heavily reduced (compare to supplemental video 1 and 2).
