# Supplementary figures and images for: Temporal window for detection of inflammatory disease using dynamic cell tracking with time-lapse MRI
Source: Sci Rep. 2018 Jun 22;8:9563. doi: 10.1038/s41598-018-27879-z (PMC6015069; doi:10.1038/s41598-018-27879-z)

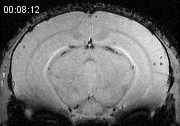

Supplement: Supplementary file 2 — Supplementary Video 1 [file 41598_2018_27879_MOESM2_ESM.gif]

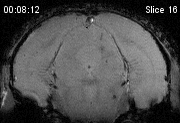

Supplement: Supplementary file 3 — Supplementary Video 2 [file 41598_2018_27879_MOESM3_ESM.gif]

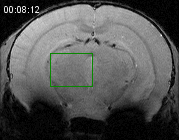

Supplement: Supplementary file 4 — Supplementary Video 3 [file 41598_2018_27879_MOESM4_ESM.gif]
